# Supplementary material for: The distribution of different intensity demanding scenarios in elite rink hockey players using an electronic performance tracking system
Source: PLoS One. 2023 Mar 9;18(3):e0282788. doi: 10.1371/journal.pone.0282788 (PMC9997928; doi:10.1371/journal.pone.0282788)
Supplement: Attachment — Submitted filename: review1.pdf [file pone.0282788.s002.pdf]

The distribution of different intensity demanding scenarios in elite rink hockey players using an electronic performance tracking system.

Abstract

Is not clear "…demanding scenario" introduction that allow to highlight the problem, emphasizing the aims studied. If author describe position of playing as a covariable of analysis (stated in the objectives), is important to understand the playing position stratification, that support the results described under ("position-dependent…interior players…exterior players"). In the methods section authors should describe the major variables of analysis, defining the units. This will support the quantitative results that are required/desirable in the abstract section. The conclusion does not stated the final application of the results, regarding the impact on the training process.

Introduction

1. The problem around the already published data is not so clear: "However, the approach used to assess average physical demand underestimates peak intensities during indoor or outdoor matches…" (line 43-45);
2. Is still unclear in what way this new methods of assessment bring new data, allowing further application. Authors need to systemize what the other instruments give and what the new data collection will allow;
3. Authors described the utility of this data for "…sports medicine practitioners…" (line 58), and I think that this is important for " sport coaches" or "sport scientists", independently of the role in the sport team;
4. This section needs revision, namely structuring what we have in literature and what this paper brings of new, before describe the final purpose of the study.

Methods

1. We suggest to adjust the design study, as a observational study, with retrospective data collection (line 82-84);
2. About the sampling/subjects, it is not clear if the data is about repetitive measures along the matches, in the same players along the years of follow-up. If so, in the design is necessary to describe this study as a observational study supported in a cohort, with a time of follow up of….years;

3. The stratification in interior and exteriors needs explanation, namely about the characteristics that fit and define the playing position (line 94-95);
4. Please reword “consent” (line 100);
5. Because physical demands were truly dependent of the opponent, how the authors take into account this on match selection?
6. Please check on the manuscript the writing word “meters” and not “metres” (line 122), check in the all manuscript;
7. The authors need to be more clear about the cutpoints or the thresholds used because of the “skating limitation” and citing studies specific of the context or highlighting this on limitation section/discussion; Futsal and basketball, despite of the indoor profile, is not the same because of the skating specificity. How authors think about this, clarifying the readers.
8. The structure of the figure 1 is not clear in the manuscript. The top 3 description do not have into account the playing position? If no, what authors think about some bias on the results?
9. Consistent statistical analysis section in contrast with some confusion in describing the general procedures. Please revise to make more clear to the readers;

Results

1. Is important to be more clear about the units of the data collection, namely if the variables presented were an average per game (supported in the 18 games collected). We know that not every players have the same opportunities about the time of playing. How authors adjust the results regarding this aspect? This have an important implication in all the dimensions of external load, and the authors describe as a team (table 2).
2. Regarding the variables collected, it seems very underestimated results, regarding the most demanding scenarios. How authors explain this (table 2)?
3. For a common reader regarding high-intensity accelerations and high-intensity decelerations, the units of theses variables were not understandable. You read as counts per something. I suggest number of accelerations/decelerations in the intensity described above (methods section) per game, adjusted for the individual playing time;
4. The authors have so many results to summarise that make the manuscript confused for the reader; I suggest a clear paragraph of the evidence from each table, conferring a clear line of results.

Discussion

1. In the first paragraph of this section authors need to be more state to the point, clarifying the answer to the research topic. Between lines 213-215, please revise the paragraph about what authors real found;
2. I suggest clear messages regarding the differences between positions and the expression of the dependent variables on the game, describing the dimensions of the load per game, in this professional level. For example, distance covered per game, by position, at high intensity? Number of high intensity accelerations and decelerations per game, per position, adjusted by playing time? The fluctuation of the load across the professional game?;
3. Authors describe as “results of table 2…” in discussion section. Please revise to “results of this study” or “in this study we observer…” (line 223); Line 236 do not use the word “figure” in this section;
4. Between lines 225-227, this results were expected to help the coaches. How? Please describe, supported on you results.
